# Exploring the genetic diversity of the IPK *Medicago* germplasm collection using GBS

**DOI:** 10.1111/plb.70170

**Published:** 2025-12-29

**Authors:** N. Devabhakthini, M. Kavka, D. Harpke, A. Himmelbach, U. Lohwasser, E. Willner, K. J. Dehmer

**Affiliations:** ^1^ Genebank Department, Satellite Collections North Leibniz Institute of Plant Genetics and Crop Plant Research (IPK) Malchow/Poel Germany; ^2^ Genebank Department, Experimental Taxonomy Leibniz Institute of Plant Genetics and Crop Plant Research (IPK) Gatersleben Germany; ^3^ Genebank Department, Genomics of Genetic Resources Leibniz Institute of Plant Genetics and Crop Plant Research (IPK) Gatersleben Germany; ^4^ Genebank Department, Resources Genetics and Reproduction Leibniz Institute of Plant Genetics and Crop Plant Research (IPK) Gatersleben Germany

**Keywords:** Alfalfa, crop wild relatives, genotyping‐by‐sequencing, population structure

## Abstract

- The genus *Medicago*, which includes the widely cultivated forage crop alfalfa, is of significant agricultural and ecological importance. Understanding genetic diversity in *Medicago* is essential for the conservation of its germplasm and its utilisation in plant breeding. This study aimed to assess the genetic diversity and population structure of the *Medicago* germplasm collection at the German Federal *Ex situ* Gene Bank.
- Genotyping‐by‐sequencing was used to analyse 1234 accessions of the Leibniz Institute of Plant Genetics and Crop Plant Research (IPK), representing 40 *Medicago* species. After filtering, a high‐quality dataset of 23,315 single nucleotide polymorphisms (SNPs) was generated.
- Our analyses revealed distinct genetic clusters corresponding to *Medicago* species and sections, with cultivated *M. sativa* L. and *M.* × *varia* Martyn clustering together with less genetic diversity compared to their wild counterparts. This reflects the shared genetic composition and extensive gene flow between *M. sativa* and *M.* × *varia*, commonly considered a hybrid between *M. sativa* and *M. falcata* L. Wild species displayed a more complex genetic structure, with polyphyletic patterns indicating higher genetic differentiation that reflects their diverse evolutionary histories and ecological adaptations.
- In conclusion, the comprehensive diversity analysis of the IPK *Medicago* collection provides valuable insights for gene bank management, targeted conservation efforts and strategic breeding initiatives.

## Graphical abstract

Genotyping‐by‐sequencing of 1234 accessions from the *Medicago* collection of the Leibniz Institute of Plant Genetics and Crop Plant Research (IPK) revealed broad genetic diversity across 40 species, providing one of the first genus‐wide insights into relationships between both cultivated and wild species of *Medicago*.

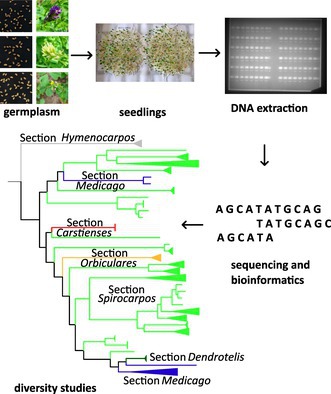

## INTRODUCTION

The leguminous genus *Medicago*, comprising a broad range of species, plays a critical role in both agriculture and ecology. The genus includes approximately 87 species, organised into 14 taxonomic sections (Small 2011). Among these species, alfalfa (*Medicago sativa* L. complex) is one of the most widely cultivated forage crops worldwide, highly valued for its nutritional quality and ability to enhance soil fertility through nitrogen fixation (Bouton 2012). Other species such as *M. lupulina*, *M. polymorpha* and *M. truncatula* Gaertn are also cultivated or used as forages on a smaller scale (Zhu *et al*. 1996). Beyond its agricultural significance, *Medicago* has become a model in plant genetics: *Medicago truncatula* is frequently utilised for research on legume biology and symbiotic nitrogen fixation (Young & Udvardi 2009). The economic and ecological importance of *Medicago* species in agriculture extends to soil conservation, as their deep root system helps to prevent soil erosion, while nitrogen fixation reduces reliance on synthetic fertilisers. Furthermore, *Medicago* species support biodiversity by providing habitats for pollinators and contributing to ecosystem stability.

Although *Medicago* species originated in the Mediterranean basin and Eurasia, they have been widely disseminated by human activity, especially *M. sativa*. Its global distribution is linked to its valuable forage attributes and adaptability (Lesins & Lesins 1979; Small 2011). Over the last few decades, modern breeding efforts have significantly advanced crop improvement often at the cost of reduced genetic diversity (Blair 1996). Studies in crops such as maize, wheat and rice have shown how breeding could have narrowed the genetic diversity in cultivated varieties (Tenaillon *et al*. 2004; Reif *et al*. 2005). This trend extends to crop species in general, as selective breeding practices promote genetic uniformity, potentially limiting adaptability to future environmental challenges (Fu 2015). For alfalfa, results are mixed: some studies suggest a narrowing of the genetic base in cultivated material compared with landraces and wild relatives (Muller *et al*. 2006), while others argue that breeding and domestication have not significantly reduced genetic variation (Li *et al*. 2012). Wild relatives and non‐cultivated accessions often retain broader genetic variation due to their adaptation to diverse environments, making them valuable for broadening the genetic base of crops. These genetic resources, safeguarded in the gene banks as cultivars, landraces and wild species, contain alleles and genes for important traits such as stress tolerance, disease resistance, yield stability and forage quality (Maxted *et al*. 1997; Tanksley & McCouch 1997; McCouch *et al*. 2013; Govindaraj *et al*. 2015). For example, many crops have benefited from the introgression of traits from wild relatives, leading to improved resilience against biotic and abiotic stresses (Tanksley & McCouch 1997; Marone *et al*. 2021). However, the phenotypic complexity and the broad genetic variability of wild relatives makes it a challenge to fully capture and characterise their genetic potential, emphasising the need for comprehensive genotypic and phenotypic analyses.

Previous research on genetic diversity in *Medicago* has primarily focused on alfalfa or a narrow subset of wild relatives. For instance, Annicchiarico *et al*. (2016) and Julier *et al*. (2018) explored genetic diversity among cultivated alfalfa populations, providing valuable insights into the genetic structure of alfalfa, but leaving gaps in our understanding of the broader diversity within the genus. Other studies, such as those by Prosperi *et al*. (2006) and Zhao *et al*. (2024), have extensively analysed diversity within specific *Medicago* species, the former using large phenotypic datasets and the latter combining phenotypic descriptors with genotyping via a DArTag panel. While these studies provide insights into genetic variation within individual *Medicago* species, comprehensive analyses of genetic diversity across a broad spectrum of species remain scarce.

Several molecular marker systems have been applied in *Medicago*, including restriction fragment length polymorphisms (RFLPs), random amplified polymorphic DNAs (RAPDs), simple sequence repeats (SSRs), amplified fragment length polymorphisms (AFLPs) and single nucleotide polymorphism (SNP) arrays, each with advantages and drawbacks. RAPD and AFLP generate many fragments but suffer from reproducibility issues, while SSRs also cover limited regions of the genome, but provide high polymorphisms. SNP arrays allow higher reproducibility on several loci in the genome, but are restricted to predefined loci from usually cultivated genotypes. With the large number of SNPs generated through genotyping‐by‐sequencing (GBS), it is possible to detect clear genetic structures even within cultivated material. In contrast, studies based on a limited number of SSR or AFLP markers detected population structures only when analysing broad diversity across wild and cultivated material, but not within breeding populations (Qiang *et al*. 2015; Annicchiarico *et al*. 2016; Herrmann *et al*. 2018). A recently developed mid‐density SNP panel (~3,000 SNPs) for alfalfa using the DArTag platform (Zhao *et al*. 2023), provides a cost‐effective and reproducible tool for cross‐programme comparisons. However, its limited marker density makes it less suitable for multispecies analyses compared with GBS. This demonstrates the advantage of GBS for resolving genetic variation at finer scales, despite the inherent missing data resulting from uneven sequencing coverage across loci and samples.

GBS enables the discovery of thousands of SNPs across large sample sets (Elshire *et al*. 2011), making it well suited for studying large germplasm collections. The GBS approach has been successfully applied in various genera to study genetic diversity and population structure, helping to support breeding strategies aimed at improving crop performance (Poland & Rife 2012). In this study, we used GBS to assess the genetic diversity and population structure of the *Medicago* germplasm collection at the Leibniz Institute of Plant Genetics and Crop Plant Research (IPK), which encompasses 1234 accessions representing 40 species. The collection reflects the diversity available in global gene banks and includes major cultivated species, close relatives and taxonomically diverse wild relatives, mostly of Mediterranean and European origin, while species with Asian centres of origin are less represented. This subset spans multiple gene pools (Maxted *et al*. 2006), with *M. sativa* as the reference point in the genus: the primary gene pool (*M. sativa* complex), the secondary gene pool (closely related species) and the tertiary gene pool (more distantly related wild taxa). The *M. sativa* complex itself is of particular importance, but also taxonomically challenging. Most experts now consider this complex as one species with subspecies such as *M. sativa* subsp. *sativa*, *M. sativa* subsp. *falcata* and *M. sativa* nothosubsp. *varia*, as well as species *M. polychroa* Grossh. and *M. hemicycla* Grossh. (Quiros & Bauchan 1988). For this study, the nomenclature is used as recorded in the IPK passport data of the Genebank Information System of IPK (GBIS, https://gbis.ipk‐gatersleben.de/gbis2i/) with *M. sativa*, *M. falcata* and *M*. × *varia* as species to ensure consistency, while acknowledging ongoing taxonomic debates.

By analysing population structure and genetic relationships, our results support gene bank management by clarifying taxonomic inconsistencies and highlighting underrepresented groups that can guide future acquisitions. For germplasm users and breeders, the dataset improves the ability to select and request accessions with defined genetic backgrounds, thereby increasing the efficiency of breeding programmes. The publicly available GBS dataset also provides a valuable foundation for downstream applications such as genome‐wide association studies (GWAS), further linking conserved diversity with traits relevant for alfalfa producers and ultimately benefiting consumers through the development of more resilient cultivars.

## MATERIALS AND METHODS

### Plant material

The entire collection of 1234 *Medicago* accessions from the IPK gene bank was used for the study. One part of the collection, comprising species used for forage, has been conserved at the Malchow/Poel site of IPK for several decades. As of February 11, 2025, the *Medicago* collection there comprises 744 alfalfa accessions from two species, namely *Medicago sativa* and *M*. × *varia*. The collection also includes 490 accessions of crop wild relatives, comprising 38 different *Medicago* species, which are preserved at the Gatersleben site of IPK. The material in the collection is of various geographic origins and represents different categories of biological status (cultivars, breeding/research material, weed/companion flora, landraces and wild accessions). Additionally, this study includes six alfalfa accessions from the National Research Institute for Agriculture, Food and Environment (INRAE, France; see also Pégard *et al*. 2023), and one *M. arborea* L. accession sourced from the seed company Neo Plantarum, Italy. These were added as external references to compare against the IPK accessions. For internal controls, six accessions from Malchow/Poel were replicated technically (identical DNA sample), and one accession was replicated biologically (DNA from different pooled individuals). A leaf sample of red clover, *Trifolium pratense* L. collected from the IPK premises, was used as the outgroup in this study. The *Medicago* species within this study were categorised into six sections based on Small (2011): *Carstienses* Kozhukarov, *Dendrotelis* (Vassilcz.) Lassen, *Hymenocarpos* Ser. in DC, *Medicago*, *Orbiculares* Urb. and *Spirocarpos* Ser. (Table 1). A comprehensive list of accessions, including their species names as recorded in GBIS (Oppermann *et al*. 2015) and the proposed species names based on this study, is available in the Table S1. Species identities were refined based on genetic clustering. Accessions that clustered closely with GBIS‐confirmed species were provisionally assigned the same species name and marked as ‘proposed’. In some cases, a species name was proposed as an alternative to the GBIS‐assigned name where clustering suggested a different taxonomic placement. Additionally, some accessions previously identified only at the genus level were labelled as ‘sp.’ (species not determined) based on GBS data and analyses, indicating a need for classical taxonomic verification through field cultivation. While genetically informed, all proposed names remain provisional and should eventually be validated using phenotypic data. Geographic regions were determined based on the country of origin information provided in the passport data from the GBIS database. Countries were then grouped into broader regions (e.g., Mediterranean, Europe, Asia) according to their geographic location.

**Table 1:** Classification of studied *Medicago* accessions into species (including proposed classification) and sections based on Small (2011).

section	*Medicago* species	number of accessions
*Carstienses*	*M. carstiensis* Jacq.	2
*Dendrotelis*	*M. strasseri* Greuter, Matthäs & Risse	1 (+ 1 biological replicate)
	*M. arborea* L.	1
*Hymenocarpos*	*M. radiata* L.	3
*Medicago*	*M. cancellata* M.Bieb.	1
	*M. cretacea* M.Bieb.	1
	*M. falcata* L.	16
	*M. glutinosa* M.Bieb.	2
	*M. hemicycla* Grossh.	1
	*M. hybrida* (Pourr.) Trautv.	1
	*M. marina* L.	1
	*M. polychroa* Grossh.	2
	*M. prostrata* Jacq.	1
	*M. sativa* L.	429 (+ 1 technical replicate)
	*M. suffruticosa* Ramond ex DC.	1
	*M*. x *varia* Martyn.	321 (+ 1 technical replicates)
*Orbiculares*	*M. orbicularis* (L.) Bartal.	4
*Spirocarpos*	*M. arabica* (L.) Huds.	7
	*M. bonarotiana* Arcang.	2
	*M. ciliaris* (L.) All.	3
	*M. coronata* (L.) Bartal.	2
	*M. disciformis* DC.	1
	*M. doliata* Carmign.	25
	*M. granadensis* Willd.	1
	*M. intertexta* (L.) Mill.	1
	*M. laciniata* (L.) Mill.	1
	*M. littoralis* Rohde ex Loisel.	2
	*M. lupulina* L.	13
	*M. minima* (L.) Bartal.	10
	*M. murex* Willd.	19
	*M. muricoleptis* Tineo	1
	*M. polymorpha* L.	217
	*M. rigidula* (L.) All.	7
	*M. rotata* Boiss.	1
	*M. rugosa* Desr.	2
	*M. scutellata* (L.) Mill.	2
	*M. tornata* (L.) Mill.	64
	*M. truncatula* Gaertn.	56
	*M. turbinata* (L.) All.	2
	*M*. sp. (not determined)	7

### DNA extraction and quantification

For each accession, 100 seeds were randomly selected and placed for germination in a controlled environment using a GS10/11 germination chamber (Flohr Instruments, Nieuwegein, The Netherlands) over 10 days with an 11‐h light period at 22°C and a 13‐h dark period at 15°C. Young cotyledons were harvested from 50 seedlings per accession, pooled and frozen at −18°C. Subsequently, the frozen samples were freeze‐dried for 24 h using an Alpha 1‐4 LD plus (Martin Christ, Osterode/Harz, Germany).

After grinding in a laboratory mixer mill (MM300, Retsch, Haan, Germany), genomic DNA was extracted from the lyophilised leaf samples using the cetyltrimethylammonium bromide (CTAB) method described by Doyle and Doyle (1990). The extraction process was conducted in a 96‐well plate format with the help of a Janus pipetting workstation (originally PerkinElmer, now Revvity; Waltham, MA, USA). Extracted DNA was suspended in 100 μl Tris‐EDTA buffer. The quality of DNA was assessed by electrophoresis on 1% agarose gels. DNA concentrations were quantified using a VICTOR Nivo multimode plate reader and the Hoechst 33258 dye (originally PerkinElmer, now Revvity; Waltham, MA, USA) and adjusted to 20 ng μl^−1^.

### GBS library preparation and sequencing

The *Pst*I and *Mse*I restriction enzyme combination (Julier *et al*. 2021) was used to digest 200 ng of genomic DNA for GBS (Zhang *et al*. 2024b) to provide genome‐wide coverage. Library preparation and individual barcoding were conducted as described previously (Zhang *et al*. 2024b) with a size fractionation of the final library (size range: 400–600 bp). In typical experiments, 250 individually barcoded samples were pooled and sequenced (single read: 118 cycles) in one lane (SP flowcell, XP‐workflow, Illumina Novaseq6000 device, Illumina, Inc., San Diego, CA, USA). For single‐read sequencing, a custom primer was used (Wendler *et al*. 2014). Per sample, approximately 2M single reads were produced. Sequencing was performed at the facilities of IPK in Gatersleben. Sequences were de‐multiplexed using the Casava pipeline 1.8 (Illumina, Inc., San Diego, CA, USA).

### Data analysis and visualisation

The raw GBS data underwent quality assessment using FastQC tools to ensure high‐quality reads. Ten samples, including eight *Medicago* accessions and two technical replicates, were removed due to low‐quality reads. After quality assessment, the final dataset comprises 1226 accessions from the IPK collection, six alfalfa samples from INRAE, one *M. arborea* sample from Neo plantarum (Italy), four technical replicates, one biological replicate and the outgroup *Trifolium pratense*. A *de novo* assembly of the GBS data was performed using ipyRAD v0.9.71 (Eaton & Overcast 2020). Restriction sites, adapters and barcodes were trimmed from all GBS sequence reads using Cutadapt within ipyRAD. Reads shorter than 35 bp after adapter removal were discarded. Two sets of output files were generated: one with all samples plus *Trifolium pratense* as outgroup (1239 samples) used for phylogenetic analysis, and another without the outgroup (1238 samples) used for principal component analysis (PCA) and population structure analysis. For both sets, the minimum sample number per locus was set to 1055, with a clustering threshold within samples of 0.85. Since most of the accessions in the collection belong to species described in literature as tetraploid, the maximum number of alleles per site was set to four. Default settings were used for other parameters including a maximum of five low‐quality bases per read and a minimum read depth of six for statistical base calling.

Diversity metrics were analysed using VCFtools (Danecek *et al*. 2011), PLINK2 (Chang *et al*. 2015) and R software (R Core Team, 2022). Initially, VCFtools was employed to generate files for heterozygosity data, allele frequencies, minor allele frequencies and missing data. Before these analyses, filtering steps were performed using PLINK2 to remove SNPs with a low call rate (<0.05) and low minor allele frequencies (<0.05). After filtering, the remaining data were converted back into VCF format for further analysis. Observed and expected heterozygosity were computed using the dplyr package (Wickham et al., 2020). Observed heterozygosity was calculated by subtracting the ratio of homozygous accessions to the total number of sites from one, while expected heterozygosity was derived similarly using the ratio of expected homozygous accessions to the total sites. Minor allele frequency (MAF) was determined by taking the minimum allele frequency among the two alleles at each locus. The frequency of missing data was assessed by analysing the proportion of missing accessions per SNP. To reduce potential biases related to ploidy and allele dosage, strict depth (e.g., minimum read depth of 6) and allele‐frequency filters were applied. Polymorphic Information Content (PIC) was calculated based on allele frequencies using the standard formula PIC = 1−∑(P_i_^2^), where P_i_ represents the frequency of the i‐th allele. All data processing utilised R with the tidyverse suite of packages, complemented by readxl (Wickham *et al*. 2019) for data import and visualisation through ggplot2 (Wickham 2016).

Phylogenetic analysis was performed using SNP data obtained from GBS‐derived variant calls. A PHYLIP‐formatted alignment was generated using the vcf2phylip.py script from a VCF file. The resulting alignment was used to infer a maximum likelihood (ML) tree using IQ‐TREE v2.1.4 (Minh *et al*. 2020). Model selection was performed using Model Finder in IQ‐TREE, which selected the best‐fit substitution model based on the Bayesian information criterion (BIC). Tree topology support was assessed using 1000 ultrafast bootstrap (UFBoot) replicates.

For population structure analysis, genotype data from a variant call format (VCF) file obtained through ipyRAD were processed using the vcf2lfmm function from the LEA package in R (Frichot & François 2015). PCA was performed using the pca function, also from the LEA package. The resulting PCA plots were generated in R using the ggplot2 package.

For model‐based Bayesian population assignment analysis, sparse non‐negative matrix factorisation (SNMF) analysis was conducted using the snmf function from the LEA package. Initially, 10 independent runs were executed for each value of K ranging from 1 to 15, with a ploidy set to 4. The optimal K value was selected based on the lowest cross‐entropy, a measure commonly used to assess the goodness‐of‐fit in SNMF analysis. The data matrix obtained from LEA for K = 4, which includes ancestral assignment frequencies, was processed using the tidyverse package in R for sorting and plotted using ggplot2. This visualisation effectively depicted different ancestral populations with colour coding.

## RESULTS

GBS sequencing generated approximately 9.78 billion raw sequence reads. The number of raw forward sequence reads per sample ranged from 611,912 to 32,829,604, with an average of 7,880,949. The assembled dataset including the *Trifolium pratense* outgroup had 5.86% missing data and consisted of 21,292 SNPs, of which 10,190 were parsimony‐informative sites. The dataset excluding the outgroup sample had 5.41% missing data and contained 26,024 SNPs, of which 12,859 were parsimony‐informative sites. After filtering the dataset excluding the outgroup, 23,315 SNP sites remained for further analysis.

### Genetic diversity and population structure

**Fig. 1:**
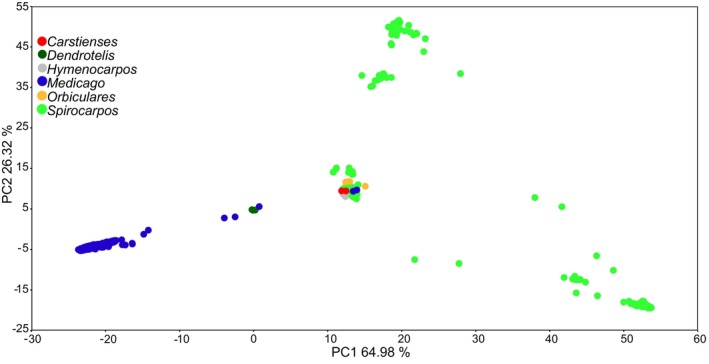
Principal component analysis (PCA) plot illustrating genetic clustering of *Medicago* accessions across six taxonomic sections.

PCA was conducted to explore the genetic diversity and population structure within the dataset. PC1 explained 64.98% of the total variance, while PC2 accounted for 26.32% (Fig. 1). The section *Medicago* with 12 species and 774 samples clustered on the negative side of PC1, forming a tight cluster with a few samples positioned in the centre and between the centre and the cluster on the left side (Fig. 1), including accessions of section *Dendrotelis*. The section *Spirocarpos* with 23 species and 444 accessions was widely distributed on the positive side of PC1, forming three main clusters: a more central cluster, a cluster towards the positive PC1 and a cluster towards the positive PC2 (Fig. 1). Section *Carstienses*, with two accessions of *M. carstiensis* Jacq., section *Hymenocarpos* with three accessions of *M. radiata* L. and section *Orbiculares* with four accessions of *M. orbicularis* (L.) Bartal. grouped within the central cluster of section *Spirocarpos* (Fig. 1; see also Fig. S3 for 3D PCA). Accessions labelled as *Medicago* sp. (species not determined; Table 1) were provisionally assigned to section‐level clusters based on their genetic placement, specifically within *Spirocarpos*.

**Fig. 2:**
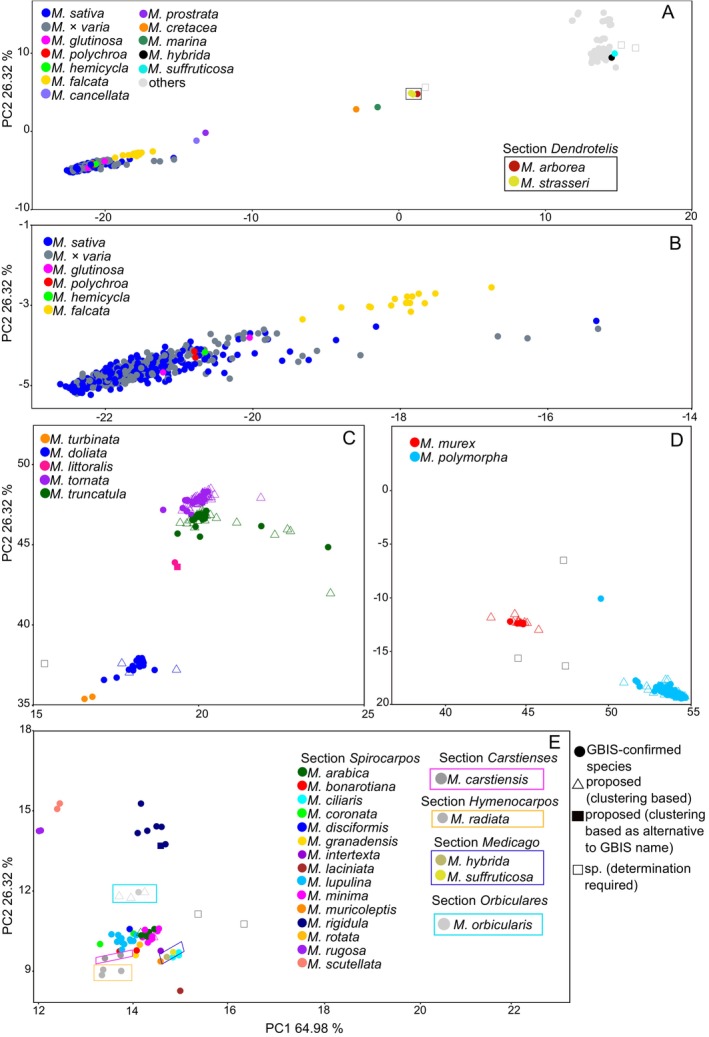
Principal component analysis (PCA) plots showing the genetic structure of species within the genus *Medicago* based on GBS data: (A) section *Medicago*; (B) section *Medicago*, with a focus on the *M. sativa* complex, including *M*. × *varia*, *M. glutinosa*, *M. polychroa* and *M. hemicycla*, where taxonomy is ambiguous; (C) section *Spirocarpos*, including *M. tornata, M. truncatula, M. turbinata and M. littoralis*; (D) section *Spirocarpos*, with a focus on *M. murex and M. polymorpha*; and (E) central cluster, primarily from section *Spirocarpos*. Species are colour coded.

An additional population structure became evident when analysing subsets of species accessions. For example, within section *Medicago*, *M. hybrida* (Pourr.) Trautv. and *M. suffruticosa* Ramond ex DC. were clustered in the central cluster, as well (Fig. 2a). *M. sativa* and *M*. × *varia* clustered closely together with *M. falcata*, along with *M. glutinosa* M. Bieb., *M. hemicycla and M. polychroa* (Fig. 2b) on the negative side of PC1, while all other species of section *Medicago* clustered in between. Within the section *Spirocarpos*, *M. tornata* (L.) Mill., *M. truncatula* and *M. littoralis* Rohde ex Loisel. formed a cluster on the positive PC2 (Fig. 2c). Below them were the species *M. doliata* Carmign. and *M. turbinata* (L.) All. Towards the positive range of PC1 were species *M. murex* Wild. and *M. polymorpha* L. (Fig. 2d). All other species (with 1–13 accessions) of the section *Spirocarpos* were clustered in the centre of the PCA (Fig. 2e).

**Fig. 3:**
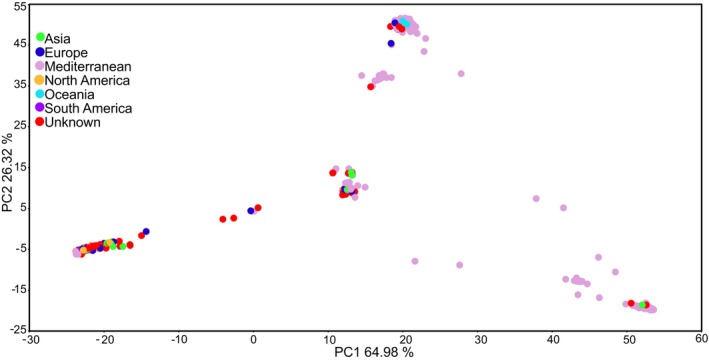
PCA plot illustrating the genetic structure of *Medicago* accessions based on geographical origin. Accessions are colour coded by region.

Figure 3 shows the regional distribution of the IPK germplasm with accessions colour coded according to their geographic origins. Accessions originating from Europe, Asia and North America form clusters, reflecting their predominantly cultivated status (*M. sativa* and *M*. × *varia*). A high number of accessions from the Mediterranean region, particularly from section *Spirocarpos*, are widely dispersed across the plot, indicating a broader genetic base. In contrast, accessions from South America and Oceania appear more scattered. Accessions with unknown (not available) origin are distributed throughout the plot.

### Phylogenetic tree

**Fig. 4:**
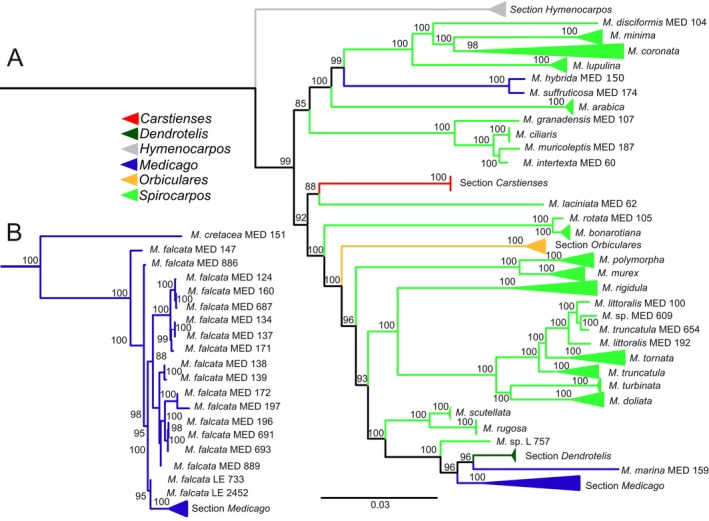
(A) Maximum likelihood (ML) phylogenetic tree of *Medicago* germplasm. Species were grouped according to their respective sections, with each section represented by a distinct colour. (B) Phylogenetic sub tree of section *Medicago*, showing *M. falcata* and its close relationship to other species in the same section.

The *Medicago* germplasm was phylogenetically analysed along with *Trifolium pratense* as an outgroup to provide a reference for evolutionary relationships within *Medicago*. The outgroup was removed from the displayed tree for better visualisation. The ML phylogenetic tree constructed from this dataset revealed defined clades (Fig. 4a,b), which align with the clustering observed in the PCA plot (Fig. 2a). Section *Medicago* formed a well‐defined clade (Fig. 4a), with *M. sativa, M*. × *varia* and *M. falcata* positioned within the same broader cluster (Fig. 4b). Additionally, *M. hybrida* and *M. suffruticosa*, although classified under section *Medicago*, clustered separately from the main *Medicago* clade, indicating that this section is not monophyletic. Section *Dendrotelis*, represented by *M. arborea* and *M. strasseri* Greuter, Matthäs & Risse, was positioned near the section *Medicago*, but does not appear as a strict sister clade. The sections *Carstienses*, *Hymenocarpos* and *Orbiculares* formed distinct monophyletic clades, positioned among section *Spirocarpos* (Fig. 4a). Unlike section *Medicago*, section *Spirocarpos* was more polyphyletic, segregating into distinct clades consistent with their PCA clustering, indicating substantial genetic divergence within this section. Several species formed well‐supported sister clades including *M. coronata* (L.) Bartal. and *M. minima* (L.) Bartal., *M. polymorpha* and *M. murex*, *M. doliata* and *M. turbinata* (Fig. 4a).

### Population structure

**Fig. 5:**
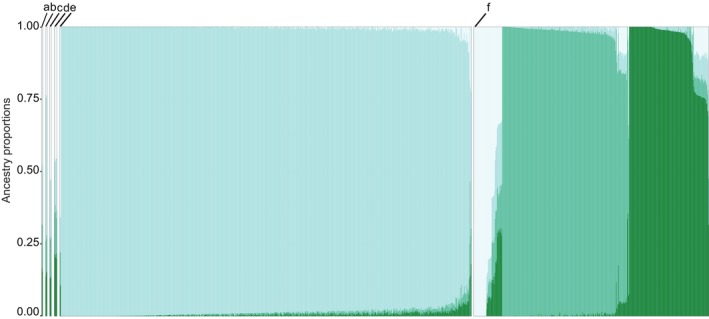
Population structure plot of 1238 accessions determined by *K* = 4. Sections are denoted in alphabetical letters. (a) *Carstienses*, (b) *Dendrotelis*, (c) *Hymenocarpos*, (d) *Orbiculares*, (e) *Medicago*, (f) *Spirocarpos*.

The structure plot (Fig. 5), determined by K = 4 using 23,315 SNPs, provides a visualisation of the genetic differentiation within each section. The sections *Medicago* and *Spirocarpos* (e and f in Fig. 5, resp.), both represented by multiple species, exhibit different patterns of genetic differentiation. In the section *Medicago* with 775 accessions which mainly include species *M. sativa*, *M*. × *varia* and *M. falcata*, the structure plot reveals 62% of accessions exhibiting >90% ancestry from a single genetic cluster. This low variation in ancestral populations indicates a relatively homogeneous genetic composition within this section, consistent with the tight clustering observed in the PCA plot (Fig. 2b). However, few samples towards the right side of the plot in the section *Medicago* (e in Fig. 5) exhibit admixture. These correspond to the less common species within section *Medicago* such as *M. cancellata* M. Bieb, *M. prostrata* Jacq. and *M. cretacea* M. Bieb. In contrast, section *Spirocarpos*, comprising mainly the species *M. polymorpha*, *M. truncatula*, *M. tornata*, *M. doliata* and *M. murex*, displays three ancestry groups in the structure plot. The first group, corresponding to the central cluster in the PCA plot, includes species such as *M. lupulina*, *M. minima* and *M. arabica*. The second group, aligning with the right cluster, primarily consists of *M. polymorpha* and *M. murex*, indicating a shared genetic background. The third group, represented by the top cluster, includes *M. tornata*, *M. truncatula* and *M. doliata*.

## DISCUSSION

### Genetic diversity

Exploring genetic diversity in the genus *Medicago* provides critical insights into the genetic differentiation of its species. Our large set of 40 species, mainly of Mediterranean/European origin, enabled a comprehensive assessment of genetic diversity and phylogeny, adding to previous studies that primarily focused on regional populations (Chen *et al*. 2020; El‐Sherif & Ibrahim 2020; Rameshknia *et al*. 2024) or cultivated varieties (Annicchiarico *et al*. 2016; Julier *et al*. 2018; Pégard *et al*. 2023).

The *Medicago sativa* species complex, which includes *M. sativa*, *M. falcata*, *M*. × *varia* and several closely related taxa, has long posed challenges for taxonomy due to extensive gene flow and overlapping traits (Fig. S4; Quiros & Bauchan 1988; Şakiroğlu & İlhan 2021). In our analyses, we adhere to the taxon names recorded in IPK's GBIS passport data to ensure consistency with official gene bank records. Only *M. sativa* and *M*. × *varia* of this complex are broadly cultivated for fodder production, yet they represent only a small fraction of the genetic diversity present in *Medicago*, as shown in our analyses. *M*. × *varia* combines genetic traits from the two parental species *M. sativa* and *M. falcata* (Kidwell *et al*. 1994; Flajoulot *et al*. 2005). Especially based on molecular methods, other authors regard *M. falcata, M. sativa, M*. × *varia* and closely related taxa such as *M. glutinosa, M. polychroa and M. hemicycla*, which are also included in our study, as a taxonomic continuum and term it *M. sativa* species complex or *M. sativa–falcata* complex (Quiros & Bauchan 1988; Şakiroğlu & İlhan 2021). The interspersing of *M*. × *varia* and the other species of the complex with *M. sativa* in our study aligns well with this view and also with previous studies showing that *M. sativa* and *M*. × *varia* are often genetically indistinguishable (Fig. S4) due to extensive gene flow and continuous introgression (Li *et al*., 2014a; Qiang *et al*. 2015). Phenotypic traits such as flower colour (purple, yellow or variegated), pod morphology (coiled vs. falcate vs. intermediate) and differences in cold hardiness have traditionally been used to differentiate taxa within the *M. sativa* complex (Şakiroğlu & İlhan 2021). However, our genomic analyses revealed that these phenotypic criteria do not always correspond to clear genetic boundaries, as distinct clusters for *M. sativa* and *M*. × *varia* were not observed. This highlights the intricacy of species delimitation within the complex and suggests that a careful re‐evaluation of taxonomic classifications in germplasm collections may be valuable. While *M*. × *varia* is expected to reflect a genetic blend of both parental species, our results indicate that *M. falcata* has played only a minor role in shaping the genetic make‐up of *M*. × *varia*, with *M. sativa* being the dominant contributor. This likely reflects the composition of our germplasm, which consists largely of cultivated material, where breeding has introgressed specific traits such as cold hardiness from *M. falcata*. Introgression in this context refers to the incorporation of only a small part of the *M. falcata* genome into the predominantly *M. sativa* background (Zhang *et al*. 2024a). Interestingly, the complete absence of private alleles in *M. falcata* (Table S2) indicates unimpeded gene flow with *M. sativa*. This challenges the species boundaries within the entire complex and raises questions about the distinctness of *M. falcata* and *M. sativa* as separate species. However, in line with our gene bank‐based approach, we retain the names as recorded in the IPK passport data (GBIS) to remain consistent with official records and avoid introducing artificial bias.

Ancient and possibly ongoing gene flow likely contributed to the mixed‐genetic nature of other *Medicago* species, too, further complicating taxonomic differentiation (Yu & Pauls 1993; Flajoulot *et al*. 2005). Morphological markers such as flower colour and pod shape have been traditionally used to differentiate these taxa, but studies have shown that these traits often fail to delineate genetic boundaries accurately (Kidwell *et al*. 1994; İlhan *et al*. 2016). Our analysis reveals that *M. carstiensis* clusters closely with *M. orbicularis* in the central PCA cluster (Fig. 2e). This close genetic proximity suggests a shared evolutionary history and a possible lineage connection between these species, which is consistent with the hypothesis discussed by Blanco‐Pastor *et al*. (2019), who referenced earlier speculation that *M. carstiensis* represents a relic species and potentially an ancestral form of *M. orbicularis*. However, both species are positioned near other taxa in the central cluster and do not form sister groups in the phylogenetic tree, indicating that their relationship may be more complex and not necessarily indicative of a direct ancestral link.

The shrubby species *M. arborea* and *M. strasseri*, traditionally classified under section *Dendrotelis*, exhibit unresolved polyphyletic origins, with no definitive parental species identified (Lesins & Lesins 1979; Rosato & Rosselló 2009). However, in our study, *M. arborea* and *M. strasseri* formed a well‐supported monophyletic clade (Fig. 4), consistent with their close clustering in the PCA (Fig. 2a). This supports earlier hypotheses that the woody growth form of *M. arborea* likely evolved from herbaceous ancestors (Steele *et al*. 2010; Smýkal *et al*. 2015).

More recently, partial hybrids between *M. sativa* and *M. arborea* have been reported, enabled by alfalfa parents producing 2n egg cells (Bingham & Irwin, 2022). Although such hybrids are rare, they show the potential for overcoming species barriers and introgressing novel alleles from woody perennials into cultivated alfalfa, thereby expanding its genetic base. Within the central PCA cluster (Fig. 2e), *M. ciliaris*, *M. intertexta* and *M. muricoleptis* exhibit a high degree of genetic similarity, consistent with previous studies highlighting their evolutionary proximity and intercrossing potential. The taxonomic distinction between *M. ciliaris* and *M. intertexta* has long been debated. Urban (1873) initially differentiated *M. ciliaris* from *M. intertexta* based on differences in dorsal suture width relative to the spine root zone. However, Lesins et al. (1971) reported successful hybridisation between *M. ciliaris* and both *M. intertexta* and *M. muricoleptis*, with F₁ hybrids displaying approximately 35% pollen fertility. This genetic compatibility likely explains their close clustering in our study and low F_ST_ values (Supplementary Table 3) suggesting substantial genetic overlap despite morphological distinctions. Positioned near *M. ciliaris*, *M. intertexta* and *M. muricoleptis* in the PCA plot (Fig. 2a) are *M. suffruticosa* and *M. hybrida*, both belonging to section *Medicago*. This suggests possible genetic affinities among these species, potentially influenced by historical gene flow or shared ancestral variation. Specifically, the central cluster (Fig. 2e) comprises species from multiple sections, including *Medicago*, *Hymenocarpos*, *Carstienses* and *Orbiculares*. The occurrence of diverse taxonomic groups in this cluster suggests that hybridisation or historical introgression events may have played a role in shaping the genetic landscape of these species. This high variation in ancestral populations reflects the polyphyletic nature and genetic diversity within the section, aligning with the dispersed pattern observed in the PCA plot (Fig. 2c–e) and the phylogenetic tree (Fig. 4), further supporting the evolutionary complexity of section *Spirocarpos* compared to the more genetically uniform section *Medicago*. These PCA patterns not only reflect taxonomic relationships but also hint at evolutionary history and local adaptation. Here, distinct clusters suggest divergence and regional adaptation, while overlaps point to historical gene flow and human‐mediated seed movement (Fig. 3).

A similar pattern is observed in the *M. littoralis—M. truncatula* complex, which has been previously recognised as a taxonomic continuum (Lesins & Lesins 1979; Small 2011). These two species are known to share gene flow (Bena 2001; Ronfort *et al*. 2006). While our PCA and phylogenetic analyses show them as closely related, the accessions identified as *M. littoralis*, *M. truncatula* and *M. tornata* appear more distinguishable in our dataset, suggesting a degree of genetic differentiation that may not fully support the continuum proposed in earlier studies. Nonetheless, this finding aligns with prior research indicating that *M. littoralis* has also interbred with *M. tornata* (Prosperi *et al*. 2006), extending the potential for gene flow beyond *M. truncatula*.

The congruence between PCA and phylogenetic analyses strengthens the validity of the proposed species names (Fig. 2c,e) and provides molecular evidence supporting their classification. However, these molecular findings will be complemented by classical taxonomic approaches, including phenotypic characterisation, to verify species identities. Although outside the scope of the current research, further phenotypic and phylogenomic analyses, along with hybridisation experiments, could help clarify the unresolved species boundaries and assess the extent of gene flow among these taxa.

### Implications for gene bank management

According to the GENESYS plant genetic resources database (https://www.genesys‐pgr.org/), approximately 71,731 accessions of *Medicago* are conserved in major gene banks worldwide. The genus *Medicago* comprises approximately 87 species divided into 14 taxonomic sections (Small 2011). In our study, we analysed 1234 accessions representing 40 Mediterranean/European species across six sections. Although this represents less than half of the total species and sections, it captures a wide genetic spectrum of the western distribution range of the genus. Several eastern sections remain underrepresented or entirely missing from the IPK collection. There are clear gaps and underrepresentation in global germplasm holdings of *Medicago* species from Central and East Asia, whereas *M. sativa* subspecies have been extensively collected throughout their range (including Central Asia). This underlines the importance of targeted collection efforts to ensure comprehensive conservation of genetic diversity. Of the 1226 *Medicago* accessions from the IPK gene bank used in this study, 12% have an unknown biostatus and 37% are cultivars, mostly *M. sativa* and *M*. × *varia*. Biostatus, along with geographic origin and botanical name, is a key component of passport data. However, incomplete or ambiguous information can hinder accurate representation of genetic relationships within collections. Our genetic analyses reveal clustering patterns that suggest shared ancestry and potential hybridisation among several *Medicago* species. Yet, taxonomic ambiguities persist, particularly within the top cluster in the PCA plot (Fig. 2c), where different classification systems assign conflicting species placements. One notable example is *M. tornata*. Lesins & Lesins (1979), in their seminal work ‘Genus *Medicago* (Leguminosae): A Taxogenetic Study’, recognised *M. tornata* as a distinct species within the section *Pachyspirae*. Similarly, the Royal Botanical Gardens, Kew, currently accepts *M. tornata* as a valid species and lists *M. italica* as its heterotypic synonym, which means they are considered the same species but were originally described from different type specimens. In contrast, Small (2011) treated *M. tornata* as a synonym of *M. italica* and did not recognise it as a separate species. Additionally, Small reclassified *Pachyspirae* as a subsection within *Spirocarpos*, grouping *M. tornata* with other species based on a broader phylogenetic interpretation. These discrepancies may indicate the presence of unusual genetic variants, natural hybrids or more probable misclassified entries. Such inconsistencies are not unexpected, as during maintenance in germplasm collections, mixes, mistakes and misidentifications can happen over time. Therefore, results involving uncertain or conflicting accessions should be taken with caution and, where possible, followed up through additional verification to confirm their taxonomic status. This highlights the need to reassess the phenotypic characteristics and taxonomic placement of these accessions. Furthermore, identifying gaps in the genetic structure of the IPK *Medicago* collection may guide the need for targeted additions, particularly in underrepresented wild species. Some species in this study were represented by only a single accession, which highlights gaps in genetic diversity presentation. While this is a limitation in the IPK collection, it reflects a broader challenge common to gene banks worldwide, particularly with crop wild relatives. In such cases, conclusions about species relationships must be drawn with caution, since single accessions cannot fully capture within‐species variation. For example, *M. hybrida* and *M. suffruticosa*, both belonging to section *Medicago*, were each represented by only one accession. Including additional accessions for these species would likely provide a more reliable assessment of their genetic placement and relationships. It is also known that these taxa are genetically distant from other members of section *Medicago*, and according to Small (2011), they have been classified in a different subsection, which may explain their distinct clustering. A brief search in GENESYS confirmed that *M. sativa* is abundantly represented in international holdings, whereas many other *Medicago* species remain underrepresented. These findings underline the need to prioritise wild relatives for acquisition, either through new collecting missions or by sourcing accessions from other collections. This would ensure that the genetic diversity within the gene bank remains robust for breeding applications aimed at enhancing resilience and adaptability (Odong *et al*. 2013; Mascher *et al*. 2019). Further efforts aligned with our findings emphasise the need for developing core collections that represent major genetic groups with minimal redundancy. Establishing core collections is generally most effective when both genotypic and phenotypic data are combined, rather than relying solely on genomic data. In our case, the comprehensive GBS dataset provides a valuable genomic foundation that could be used in the future to define an alfalfa core collection. Such a collection would ideally integrate SNP diversity with phenotypic evaluations across multiple environments (e.g., biomass, winter survival, disease resistance), as demonstrated in other crops and forages (Tanksley & McCouch 1997; Keep *et al*. 2020; Phogat *et al*. 2021). Through this approach, we aim not only to conserve but also to optimise the accessibility of genetic diversity within the collection, ultimately supporting future forage breeding initiatives. Additionally, this study contributes to taxonomic curation by proposing species names for 211 out of 218 accessions previously identified only at the genus level via a clustering close to genetically confirmed ones. Furthermore, we could validate identifications for 1,005 out of 1,008 accessions. As detailed in the Materials and Methods and Supplementary Table 1, these proposed names help prioritise accessions for future phenotypic verification through field cultivation. This stepwise approach allows curators to focus resources on ambiguous cases. All proposed names remain provisional and should be confirmed through classical taxonomic methods in subsequent curation efforts.

### Utilising the diversity for breeding programmes

Cultivated *M. sativa* has experienced a reduction of 9%–30% in genetic diversity compared to its wild counterparts, as revealed through genome‐wide polymorphism analyses, likely reflecting domestication bottlenecks and repeated selection during breeding (Muller *et al*. 2006). However, subsequent genome and transcriptome‐wide analyses revealed that much of the variation present in wild populations is still retained within cultivated germplasm. For instance, Li *et al*. (2012) identified a lower prevalence of SNPs in cultivated *M. sativa* but noted that most genetic variation observed in wild accessions persists in domesticated forms. More recently, Medina *et al*. (2025) also demonstrated that a high proportion of SNPs segregate within or among elite cultivars from commercial breeding programmes, confirming that substantial genetic variation remains available for breeding. Collectively, these studies suggest that while domestication has contributed to a partial reduction in genetic diversity, cultivated *M. sativa* still retains a broad genetic base. This underscores the importance of conserving genetically diverse and representative collections, including crop wild relatives, to safeguard allelic variation for breeding programmes. Particularly in wild species, the genetic diversity observed within our study reveals genetic dispersion among species, such as *M. polymorpha, M. murex* and *M. truncatula* in the section *Spirocarpos* (Fig. 4). This genetic differentiation, as reflected in pairwise F_ST_ values (see Fig. S2 and Table S3), highlights their adaptation to various ecological niches and suggests that wild relatives may play a crucial role in broadening the genetic base available for breeding programmes, offering valuable traits for developing improved germplasm and cultivars. Crosses involving *M. sativa* and other species of the genus have been explored: Hybrids between *M. sativa* and *M. papillosa* were successfully generated, but ovule‐embryo culture was required to recover fertile hybrids (McCoy & Smith 1986). Similarly, somatic hybrids between *M. rugosa* and *M. scutellata* were produced via protoplast fusion (Mizukami *et al*. 2006). These hybrids faced challenges such as genomic instability and loss of chromosomes during vegetative proliferation, but demonstrated the potential for transferring traits between these species. Additionally, hybrids have successfully been generated in crosses between *M. sativa* and *M. arborea*, exhibiting traits such as increased drought tolerance, improved habit and larger seed size, emphasising the value of wild species as genetic reservoirs for resilience traits (Humphries *et al*. 2021; Bingham & Irwin, 2022). Furthermore, hybrids derived especially from *M. sativa* and *M. arborea* have demonstrated positive heterosis in yield performance, particularly under subtropical and arid conditions, highlighting the potential of wild species in changing climatic conditions (Irwin & Bingham 2023).

Overlapping clusters based on different geographical origins indicate a shared genetic background likely resulting from seed exchanges and human‐mediated movement of plant material across regions, leading to substantial gene flow and admixture (Ellstrand & Rieseberg 2016). This genetic connectivity suggests that breeding programmes can access a broad pool of genetic material from various accessions, regardless of their geographic origin. The presence of shared genetic backgrounds across accessions categorised as breeding material, cultivars, landraces and wild plants supports the notion that desirable traits may be present across diverse germplasm, offering breeders a versatile foundation for selection.

### Genotyping‐by‐sequencing as a tool for diversity analyses in *Medicago*

Advances in next‐generation sequencing and genome‐wide screening have made GBS an effective tool for SNP discovery and genotyping in crop species. However, taxonomic complexity, differing ploidy levels, as well as past and recent introgressions in *Medicago* species present unique challenges in GBS studies. The *Pst*I–*Mse*I restriction enzyme combination, previously validated in *M. sativa* by Julier *et al*. (2021), generated a high‐quality dataset of 23,315 SNPs with less than 5.41% missing data per locus. Although this number is lower than the 89,216 SNPs reported by Pégard *et al*. (2023), their study focused exclusively on *M. sativa*, where conserved restriction sites across individuals likely contributed to higher locus recovery and deeper coverage. In contrast, our multispecies dataset spans a broader phylogenetic range, which can reduce the number of shared loci and limit overall SNP yield. Earlier studies using GBS in *Medicago* reported substantially fewer SNPs, typically between 1,500 and 3,591. This is largely due to the smaller number of genotypes analysed in those studies. For example, Annicchiarico *et al*. (2016) analysed 11 landraces, Li *et al*. (2014b) included 384 F₁ progenies with two parents and Zhao *et al*. (2024) examined 194 accessions but recovered only ~3,000 SNPs before filtering. Direct comparisons across studies are complicated by technical and biological factors. Differences in sequencing depth, read length and the randomness of restriction enzyme site distributions, as well as variation in sampling strategies, library preparation protocols, species complexity and bioinformatics pipelines are all likely to contribute to the observed differences in SNP recovery. In our study, the application of stringent filtering parameters helped reduce common issues associated with large SNP datasets, particularly missing data and low‐confidence loci, as recommended by Heslot *et al*. (2013) and Li *et al*. (2015). The bulk sampling strategy applied in our study reliably estimated allele frequencies in heterozygous populations, as demonstrated in previous research (Rocher *et al*. 2015; Annicchiarico *et al*. 2016; Julier *et al*. 2018). Although the downstream bioinformatics remains complex and time intensive, this approach is particularly well suited for large‐scale genetic diversity assessments in outcrossing forage species, where it enables efficient and cost‐effective genotyping within large collections. While bulk sampling tends to represent population‐level averages and may overlook outliers or rare alleles (Carelli *et al*. 2009), we attempted to mitigate this limitation by using high sequencing coverage. Our comparative analysis using individual plant genotyping (Fig. S1) revealed that both strategies captured a comparable number of loci. Specifically, bulk samples retained 12,257 loci post‐filtering, and individual replicates showed minimal variation in locus recovery, confirming the reproducibility of both sampling approaches. Nevertheless, while bulk sampling efficiently captures prevalent alleles, it may still underrepresent rare alleles and subtle genetic variation. For studies requiring higher resolution or fine‐scale population structure analysis, individual genotyping remains a valuable complementary approach to overcome this limitation. To further enhance SNP discovery and ensure comprehensive representation of the collection, we applied a de novo SNP calling pipeline as used in previous *M. sativa* studies (Annicchiarico *et al*. 2015; Biazzi *et al*. 2017; Li et al. 2014b). This decision was driven by the limitations of available reference genomes. While high‐quality assemblies exist for *M. truncatula* and *M. sativa* (Chen *et al*. 2020; Shen *et al*. 2020; Long *et al*. 2022), they do not encompass the full species diversity present in the IPK *Medicago* collection. Relying on a single reference genome increases the risk of reference bias and may exclude species‐specific genomic regions (Stetter & Schmid 2017). Additionally, our study confirms SNP consistency via the inclusion of technical and biological duplicates. As expected, they were clustered very closely in both PCA and phylogenetic analyses, validating the reliability of our genotyping approach and SNP detection. Euclidean distances between replicate pairs based on their PCA coordinates were calculated. These values are markedly lower than distances observed between randomly selected unrelated accessions (see Table S4), supporting our genotyping results.

## CONCLUSION

Our study reveals substantial genetic diversity across 1234 accessions representing 40 *Medicago* species, with distinct genetic clusters corresponding to taxonomic units like species and sections. Cultivated material (*M. sativa* and *M*. × *varia*) appeared genetically less diverse and tended to cluster more tightly, although this pattern may partly reflect the high number of accessions sampled from only two species. In contrast, wild material exhibited broader genetic variability and greater dispersion. Our results can be applied in gene bank management as well as in the development of strategic breeding programmes. In addition, complementation of these genotypic data with phenotypic characterisations and evaluations will further valorise the IPK *Medicago* collection.

## AUTHOR CONTRIBUTIONS

**ND:** Data curation, formal analysis, investigation, methodology, visualisation, writing—original draft. **MK:** supervision, validation, visualisation, writing—review and editing. **DH:** supervision, validation, visualisation, writing—review and editing. **AH:** Data curation, writing—review and editing. **UL:** writing—review and editing. **EW:** conceptualisation, funding acquisition, resources, supervision. **KJD:** conceptualisation, funding acquisition, project administration, resources, supervision, writing—review and editing.

## FUNDING INFORMATION

This research was funded by the Federal Ministry of Food and Agriculture (BMEL) based on a decision of the parliament of the Federal Republic of Germany via the Federal Office for Agriculture and Food (BLE) (Grant Code 2818EPS035).

## CONFLICTS OF INTEREST

The authors declare neither financial nor non‐financial competing interest.

## Supporting information


**Fig. S1.** PCA plot illustrating the genetic clustering of *M. sativa* and *M*. × *varia* accession replicate samples derived from single‐plant genotyping (triangles) within all accessions and their corresponding pooled samples (circles).
**Fig. S2.** Pairwise genetic differentiation F_ST_ values among different *Medicago* species. The heatmap visually represents the degree of genetic differentiation, where higher values (depicted in warm colours) indicate greater genetic divergence, while lower values (depicted in cool colours) suggest closer genetic relationships. Squares indicate species which require further determination. Filled squares represent proposed species names alternative to the GBIS names. Triangles indicate proposed species names based on clustering patterns observed in the PCA analysis.
**Fig. S3.** Three‐dimensional PCA of *Medicago* accessions, showing clustering of sections along PC1, PC2 and PC3.
**Fig. S4.** Phylogenetic tree of representative accessions from the *M. sativa* complex, including *M. hemicycla* and *M. polychroa*. Branch tip colours indicate species identity: blue = *M. sativa*, red = *M. polychroa*, light green = *M. hemicycla*, grey = *M*. × *varia* and yellow = *M. falcata*.

**Table S1.** *Medicago* species designation.
**Table S2.** Shared and private alleles between *M. falcata* and *M. sativa*.
**Table S3.** Pairwise F_ST_ values among *Medicago* species.
**Table S4.** Euclidean distances between technical, biological and randomly selected *Medicago* sample pairs based on PCA coordinates.

## Data Availability

The sequence data for this study have been deposited in the European Nucleotide Archive (ENA) at EMBL‐EBI under accession number PRJEB89658 (https://www.ebi.ac.uk/ena/browser/view/PRJEB89658).
