# Supplementary material for: Exploring the genetic diversity of the IPK Medicago germplasm collection using GBS
Source: Plant Biol (Stuttg). 2025 Dec 29;28(2):345–58. doi: 10.1111/plb.70170 (PMC12884036; doi:10.1111/plb.70170)
Supplement: Supplementary file 1 — Fig. S1. PCA plot illustrating the genetic clustering of M. sativa and M. × varia accession replicate samples derived from single‐plant genotyping (triangles) within all accessions and their corresponding pooled samples (circles). Fig. S2. Pairwise genetic differentiation FST values among different Medicago species. The heatmap visually represents the degree of genetic differentiation, where higher values (depicted in warm colours) indicate greater genetic divergence, while lower values (depicted in cool colours) suggest closer genetic relationships. Squares indicate species which require further determination. Filled squares represent proposed species names alternative to the GBIS names. Triangles indicate proposed species names based on clustering patterns observed in the PCA analysis. Fig. S3. Three‐dimensional PCA of Medicago accessions, showing clustering of sections along PC1, PC2 and PC3. Fig. S4. Phylogenetic tree of representative accessions from the M. sativa complex, including M. hemicycla and M. polychroa. Branch tip colours indicate species identity: blue = M. sativa, red = M. polychroa, light green = M. hemicycla, grey = M. × varia and yellow = M. falcata. [file PLB-28-345-s002.pdf]

# Supplementary Figures

for

**Exploring the genetic diversity of the IPK *Medicago* germplasm collection using GBS**

Nagarjun Devabhakthini, Mareike Kavka, Dörte Harpke, Axel Himmelbach, Ulrike Lohwasser, Evelin Willner, Klaus J. Dehmer

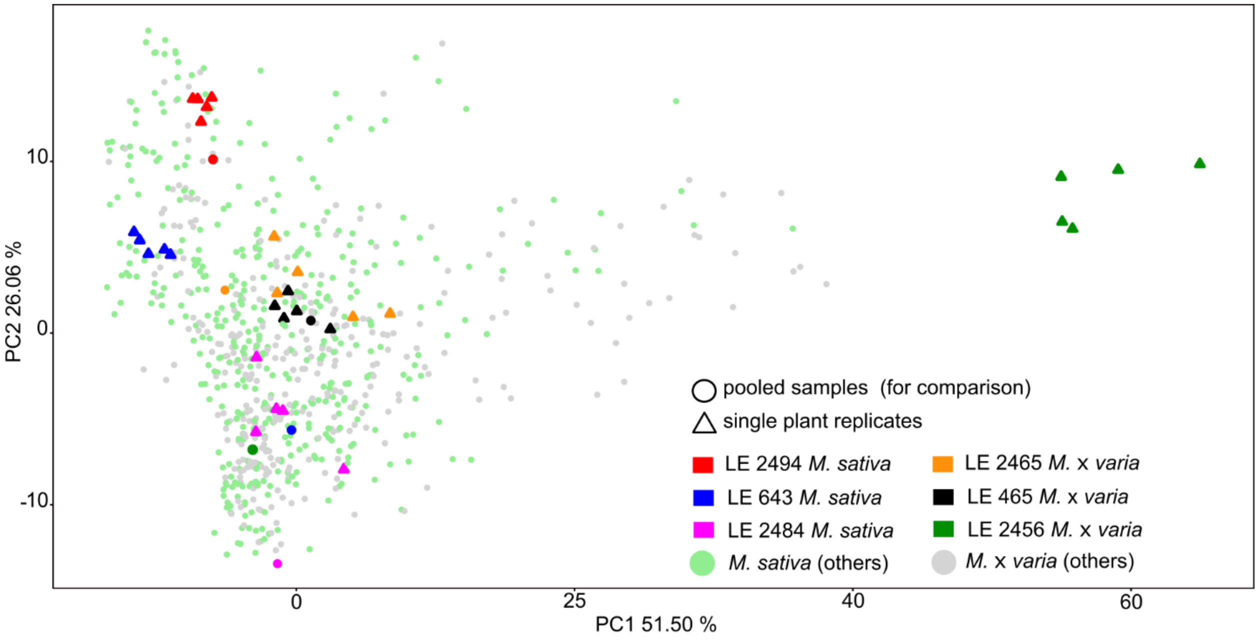


**Figure S1:** PCA plot illustrating the genetic clustering of *M. sativa* and *M.* x *varia* accessions replicate samples derived from single-plant genotyping (triangles) within all accessions and their corresponding pooled samples (circles).

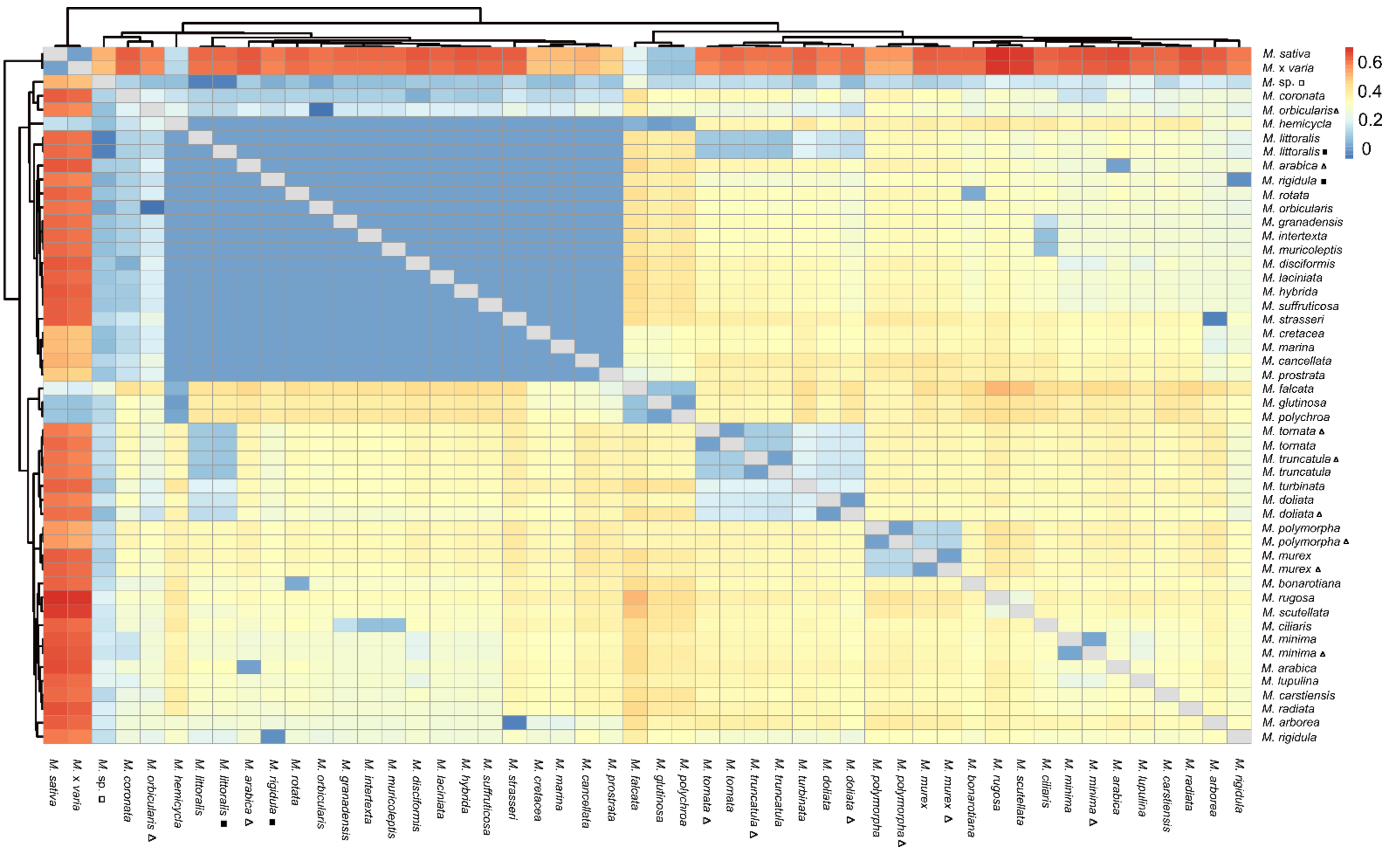


**Figure S2:** Pairwise genetic differentiation $F_{ST}$ values among different *Medicago* species. The heatmap visually represents the degree of genetic differentiation, where higher values (depicted in warm colours) indicate greater genetic divergence, while lower values (depicted in cool colours) suggest closer genetic relationships. Squares indicate species which require further determination. Filled squares represent proposed species names alternative to the GBIS names. Triangles indicate proposed species names based on clustering patterns observed in the PCA analysis.

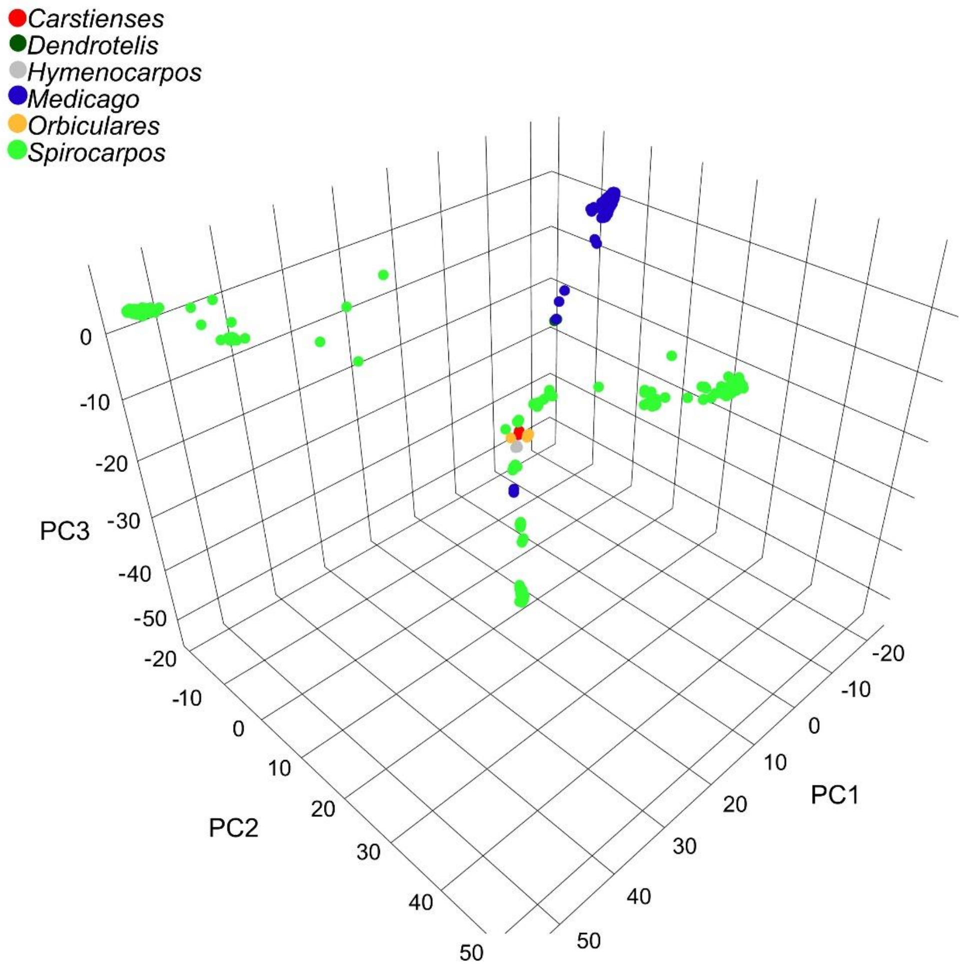


**Figure S3:** Three-dimensional PCA of *Medicago* accessions, showing clustering of sections along PC1, PC2, and PC3.

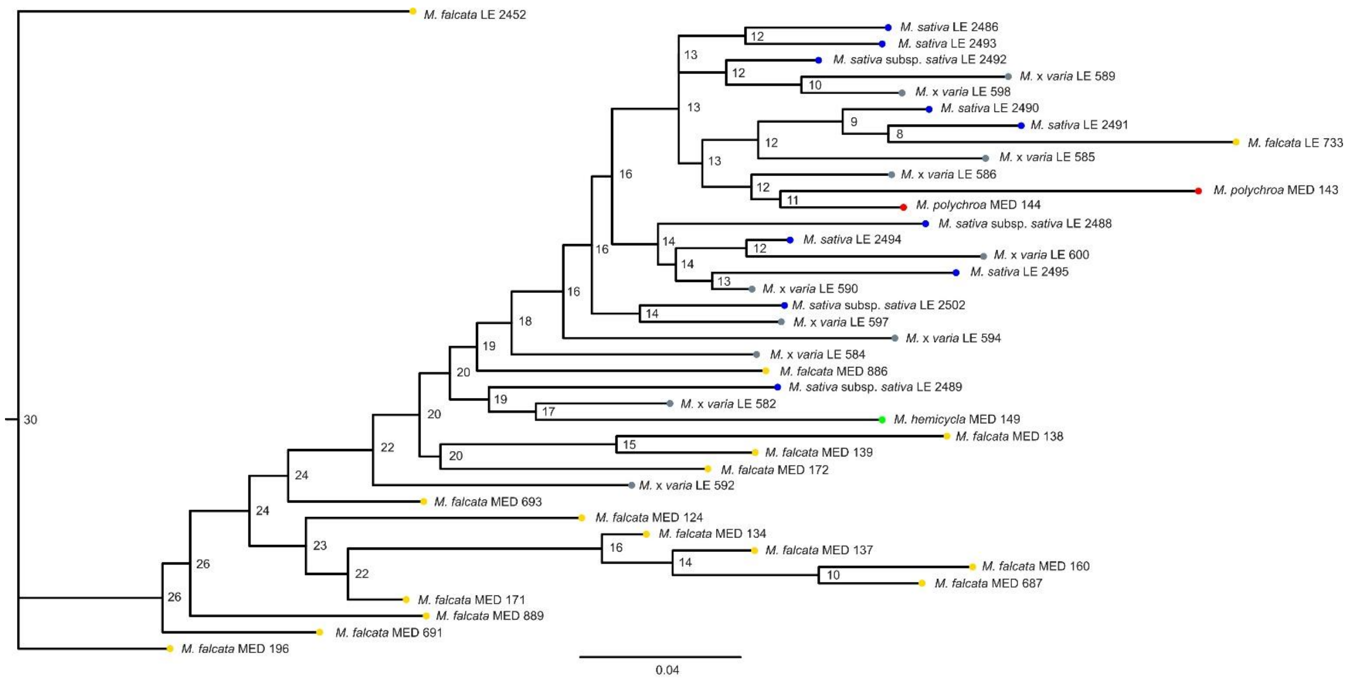


**Figure S4:** Phylogenetic tree of representative accessions from the *M. sativa* complex including *M. hemicycla* and *M. polychroa*. Branch tip colors indicate species identity: blue = *M. sativa*, red = *M. polychroa*, light green = *M. hemicycla*, grey = *M.* x *varia*, and yellow = *M. falcata*.
